# Image Dehazing Using LiDAR Generated Grayscale Depth Prior

**DOI:** 10.3390/s22031199

**Published:** 2022-02-05

**Authors:** Won Young Chung, Sun Young Kim, Chang Ho Kang

**Affiliations:** 1 Department of Aerospace Engineering, Automation and System Research Institute, Seoul National University, Seoul 08826, Korea; 26553@snu.ac.kr; 2 School of Mechanical Convergence System Engineering, Kunsan National University, Gunsan 54150, Korea; 3 Department of Mechanical System Engineering (Department of Aeronautics, Mechanical and Electronic Convergence Engineering), Kumoh National Institute of Technology, Gumi 39177, Korea

**Keywords:** dehazing, LiDAR, scattering coefficient, depth

## Abstract

In this paper, the dehazing algorithm is proposed using a one-channel grayscale depth image generated from a LiDAR point cloud 2D projection image. In depth image-based dehazing, the estimation of the scattering coefficient is the most important. Since scattering coefficients are used to estimate the transmission image for dehazing, the optimal coefficients for effective dehazing must be obtained depending on the level of haze generation. Thus, we estimated the optimal scattering coefficient for 100 synthetic haze images and represented the distribution between the optimal scattering coefficient and dark channels. Moreover, through linear regression of the aforementioned distribution, the equation between scattering coefficients and dark channels was estimated, enabling the estimation of appropriate scattering coefficient. Transmission image for dehazing is defined with a scattering coefficient and a grayscale depth image, obtained from LiDAR 2D projection. Finally, dehazing is performed based on the atmospheric scattering model through the defined atmospheric light and transmission image. The proposed method was quantitatively and qualitatively analyzed through simulation and image quality parameters. Qualitative analysis was conducted through YOLO v3 and quantitative analysis was conducted through MSE, PSNR, SSIM, etc. In quantitative analysis, SSIM showed an average performance improvement of 24%.

## 1. Introduction

Haze is a phenomenon in which the visible distance is reduced due to dust, smoke particles, and polluting particles in the atmosphere. Particles in the atmosphere scatter light; thus, images obtained in these environments decrease contrast and, eventually, deteriorate visibility. Recently developed automatic navigation systems rely heavily on vision sensors [1]. If the input image is in poor condition, the overall system will suffer. Therefore, dehazing technology, which can obtain clear images, can benefit systems such as image classification [2,3,4,5], image recognition [6,7,8,9,10], visual odometry [11,12], and remote sensing [13,14,15].

Currently, the most commonly used sensors for robots and vehicles include light detection and ranging (LiDAR) and camera. The sensors allow the performance of visual odometry, LiDAR odometry, SLAM, autonomous navigation, etc. For these purposes, they can be used as visual-only [11,12,16,17], LiDAR-only [18,19], or fused [20]. When the LiDAR and visual are fused, the two sensors are used complementarily to increase robustness of the system [20]. Even with the increase in robustness, damage to the resulting values can occur if the input data obtained from the sensors are inherently in poor condition. Therefore, it is necessary to make quality input data to prevent this degradation.

**Figure 1:**
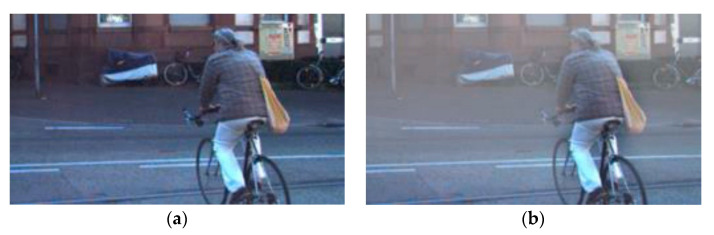
Clear image and hazy image: (**a**) clear image; (**b**) hazy image.

We can attach cameras to numerous platforms such as vehicles, drones, and robots to get image data. If the weather allows, we can get clear images such as Figure 1a. In this case, dehazing is unnecessary for effective vision-based processes. However, in the event of haze due to smoke or fine dust in the atmosphere, such as in Figure 1b, utilizing such processes becomes challenging [21]. Therefore, the contrasts from images obtained in a hazed environment should be enhanced.

Each image pixel value in hazy images can be expressed with atmospheric scattering model, a linear combination of the pixel values from actual image, transmission image, and airlight [22,23,24]. Airlight and transmission images are required to perform scene radiance recovery through the scattering coefficient model.

Traditional algorithms use color attenuation prior [25] and dark channel prior [26] to create a transmission image, whereas recent research uses deep learning to perform dehazing. The proposed method succeeds traditional methods, with its contribution in utilizing depth image and scattering coefficient to perform dehazing.

Existing methods for obtaining depth images include using stereo camera or depth camera. More recent research adopts deep learning in obtaining depth images from monocular images through training of existing depth images [27]. In this paper, the depth image is obtained by 2D projection of LiDAR point cloud. In this way, by obtaining a depth image through LiDAR and performing dehazing on vision data, we would like to propose a more complementary and robust LiDAR–vision fusion system.

Our contribution is as follows: (1) Proposal of a depth image-based dehazing technique available in LiDAR–vision fusion systems; (2) Proposal of a scattering coefficient estimation technique through the DCM-scattering coefficient model.

An outline of the paper is as follows. Section 2 outlines the theoretical background of dehazing and the related works applied to the proposed method. Section 3 outlines the overall description of the proposed method, and Section 4 summarizes the analysis of the simulation results obtained through the proposed method. Finally, Section 5 briefly describes the conclusions, the limitations of the proposed method, and the future works for improving the limit.

## 2. Image Dehazing

### 2.1. Related Works

Image dehazing has always been a popular method to obtain clear images for image processing. Due to its popularity, numerous methods of dehazing have been proposed. Assumption-based and prior-based methods are typically used. Tan et al. [28] found that the contrast was higher for images without haze than those with haze. Thus, Tan et al. [28] performed single image dehazing by maximizing the local contrast for single images. Fattal et al. [29] discovered that pixels of image patches typically exhibit a one-dimensional distribution, and used it to recover the scene transmission. Huo et al. [30] performed dehazing with the white balance algorithm and the atmospheric illuminance prior. Zheng et al. [31] performed dehazing with patch adaptive structure decomposition and multi-exposure image fusion. He et al. [26] uses the assumption that pixels without haze tend to have a meager intensity value for at least one channel out of three RGB channels.

He defined this channel as the dark channel and used it to create a transmission image. The method from He is the most widely used and considered a standard in dehazing. Zhu et al. [25] proposed a color attenuation prior-based method to generate a depth image from a hazy image. Here, the transmission image used to obtain the dehazed image was obtained through the relationship between the depth image and the transmission image.

Recently, learning-based methods have also been proposed [32,33,34,35,36,37]. Cai et al. [32] proposed an end-to-end dehazing using a convolutional neural network (CNN) model. This was done by estimating the transmission image using the BReLU and Maxout activation functions. Ren et al. [33] performed dehazing by using multiscale CNN to estimate the transmission image. In addition, Li et al. proposed AOD-NET [34] and Dehze-cGAN [35] using the generative adversarial network.

In this paper, the proposed method uses a depth image to obtain the transmission image. During the process, the required scattering coefficient is obtained by estimating through the relationship between dark channel and scattering coefficient. Then dehazing is performed using the obtained transmission image.

### 2.2. Atmospheric Scatterming Model

**Figure 2:**
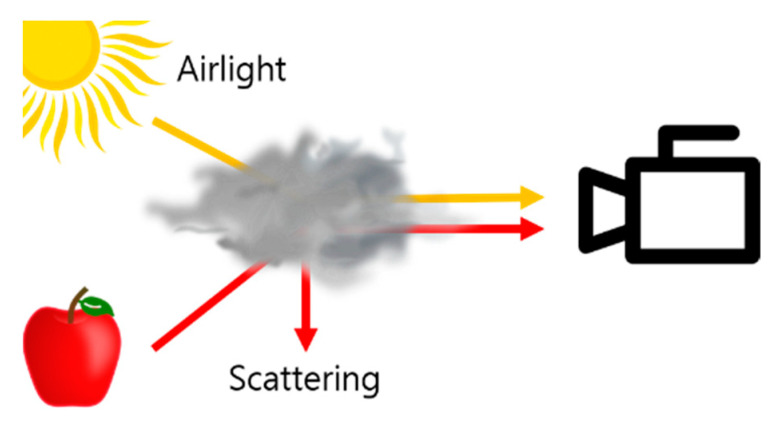
Atmospheric scattering model.

Due to the light scattered by the atmosphere, and the atmospheric light, the hazy image looks blurry, as shown in Figure 2. This phenomenon can be explained by Equation (1) [22,23,24].

(1) I(x)=J(x)t(x)+A(x)(1−t(x))

where x represents a two-dimensional vector, comprised of the position of each pixel in the image. J(x) is an image before being distorted by haze, which is the ultimate result we want to obtain through the above equation. I(x) is the hazy image and t(x) is the transmission image, representing the proportion of light that reaches the camera through the atmosphere. A is airlight, and it is assumed that all pixels in the image have the same value.

From Equation (1), we can see that the information I(x) from the camera is lost as the actual information J(x) and the signal reflected from the target pass through the atmosphere, leaving only the J(x)t(x) level. In addition, A(x)(1−t(x)) caused by atmospheric light sources is mixed, resulting in haze, shown in Figure 1b.

Through Equations (1) and (2) can be derived to obtain dehazed image J(x):

(2) $$J(x)=\frac{I(x)-A(x)(1-t(x))}{t(x)}$$

As the distance between the object and the camera increases, the atmosphere between the camera and the object becomes thicker. In other words, when the scattering coefficient is a constant, the further the distance, the worse the haze becomes. Through this relationship, Equation (3) can be obtained.

(3) $$t(x)=e^{-\beta d(x)}$$

where β is the scattering coefficient, which is a constant indicating the level at which light is scattered due to fine particles. d(x) is the depth image, x is the distance between the target and the observer to the pixel, and t(x) is the transmission image.

### 2.3. Dark Channel Prior

According to He et al. [26], for pixels without haze, most of the three RGB channels tend to have low values for at least one channel. For channels exhibiting this tendency, the author defines it as the dark channel, hence, such prior using them referred to as the dark channel prior. The following Equation (4) defines the dark channel for image J(x):

(4) $$J^{dark}(x)=\underset{c\in \{r,g,b\}}{\min}(\underset{y\in \Omega (x)}{\min}(J^{c}(y)))$$

where $J^{dark}$ is the dark channel of image and $J^{c}(y)$ is the color channel of the pixel x of image. Ω(x) is a set of pixels within a specific range centered on pixel x.

The equation shows that the value of the dark channel, which corresponds to a specific pixel x of the image, is the smallest value of the pixels around x. In He et al. [26], the pixel which has a small dark channel value is primarily one of the following three cases: (1) shadow area caused by object; (2) colorful object or surface; or (3) black or dark object or surface.

Dark channel images generally have small pixel values because natural images without haze are darkened by color or shadow [26]. However, if haze occurs, these objects will become blurry and invisible, resulting in a large dark pixel value and a white dark channel image. Based on these notions, we can identify the haze intensity of the image.

### 2.4. Guided Filter

The guided filter uses a guide image as an edge-preserving smoothing filter to perform smoothing without distorting key information of the entered image [38]. The filter assumes that the output images can be modeled linearly with guide images and linear coefficients.

(5) $$q_{i}=a_{k}I_{i}+b_{k},\;\forall i\in \omega_{k}$$

(6) $$q_{i}=p_{i}-n_{i}$$

where $q_{i}$ is the output image, $I_{i}$ is the guide image, and $a_{k}$ and $b_{k}$ are linear coefficients constant within $\omega_{k}$. Since the linear coefficient $\left(a_{k},b_{k}\right)$ in Equation (5) must be determined, Equation (5) is modeled as Equation (6) where $p_{i}$ is the input image and $n_{i}$ is the noise in the image. Then, we define the cost function to obtain linear coefficients through finding a solution that minimizes the cost function. The cost function is defined in Equation (7).

(7) $$E(a_{k},b_{k})=\sum_{i\in \omega_{k}}({\left(a_{k}I_{i}+b_{k}-p_{i}\right)}^{2}+\varepsilon a_{k}^{2})$$

where ε is the regularization parameter, which prevents $a_{k}$ from growing infinitely. The solution to minimize Equation (7) is Equations (8) and (9).

(8) $$a_{k}=\frac{\frac{1}{\left|\omega \right|}\sum_{i\in \omega_{k}}I_{i}p_{i}-\mu_{k}{\bar{p}}_{k}}{\sigma_{k}^{2}+\varepsilon }$$

(9) $$b_{k}={\bar{p}}_{k}-a_{k}\mu_{k}$$

where $\sigma_{k}^{2},\;\mu_{k}$ is the variance and mean of $I_{i}$ within the $\omega_{k}$ region. |ω| is the number of pixels in the region $\omega_{k}$. Lastly, ${\bar{p}}_{k}$ is defined in Equation (10).

(10) $${\bar{p}}_{k}=\frac{1}{\left|\omega \right|}\sum_{i\in \omega_{k}}p_{i}$$

After obtaining linear coefficients via Equations (8) and (9), the output image $q_{i}$ can be calculated. In this equation, the size of the region $\omega_{k}$ and ε affect edge-preserving and smoothing the output image.

## 3. Image Dehazing Based on LiDAR Generated Grayscale Depth Prior

**Figure 3:**
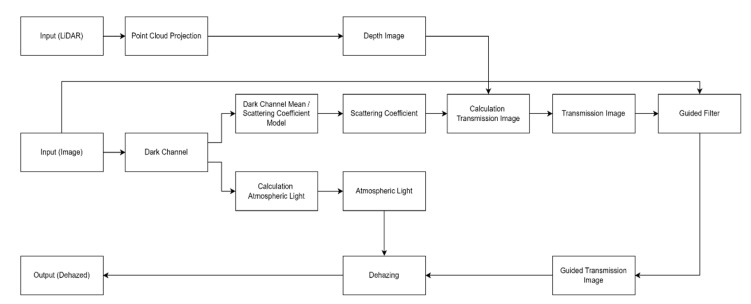
Flowchart of the proposed method.

The structure of the proposed dehazing method is shown in Figure 3. First, hazy image and point cloud are used as input data. Through a relationship in Section 3.1, the point cloud is projected and converted into a depth image. When projecting the point cloud, the point cloud of the LiDAR must be projected within the camera frame through the calibration of the camera and LiDAR. Thereafter, the scattering coefficient is estimated through a relationship in Section 3.2 by using the image with haze as an input image. The dark channel image used in Section 3.2, obtained from the hazy image, is also used to estimate the atmospheric light. Finally, the transmission image is estimated through the depth image and the scattering coefficient, and after refining the transmission image by applying the guided filter in Section 3.3, the dehazing is performed according to Equation (13).

### 3.1. Point Cloud Projection

**Figure 4:**
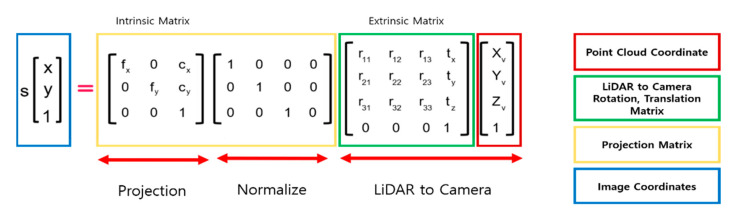
Point cloud 2D projection.

In this study, synthetic haze image generation and verification of dehazing algorithm are performed using a KITTI dataset [39]. In order to generate depth images required for the dehazing algorithm, the point cloud of the KITTI dataset was projected into an image [37]. Using the calibration data from the dataset, the projection, rotation, and translation matrices can be obtained, and the point cloud in 3D format projects into 2D through the relationship shown in Figure 4.

First, when a point in the 3D space is represented as ${\left[X,Y,Z,1\right]}^{T}$, its position on the 2D image is expressed in ${\left[x,y,1\right]}^{T}$ where X, Y, and Z refer to the coordinates of a point cloud in the world frame, and x and y are image pixel coordinates in the camera frame. To perform a projection, points in the 3D space belonging to the world frame should be represented within the camera frame. This can be expressed by multiplying the world frame’s rotation with the matrix extrinsic matrix for translation.

**Figure 5:**
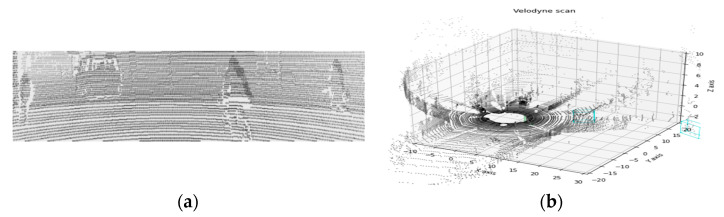
Result of point cloud projection: (**a**) projected 2D point cloud; (**b**) 3D point cloud.

Then, ${\left[X,Y,Z,1\right]}^{T}$ can be projected onto a two-dimensional plane by normalizing the obtained values and multiplying them by the intrinsic matrix containing focal length $\left(f_{x},f_{y}\right)$ and principle points $\left(c_{x},c_{y}\right)$. Figure 5 is a 2D depth image obtained through point cloud projection.

**Figure 6:**
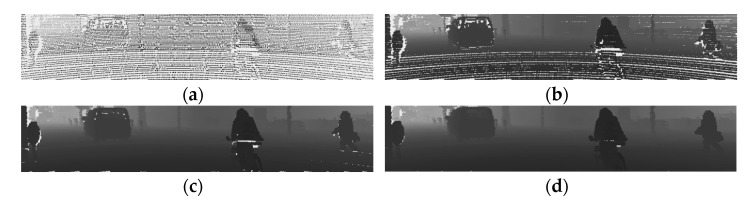
Generated grayscale depth image with different size of point cloud: (**a**) size = 1; (**b**) size = 3; (**c**) size = 5; (**d**) size = 7.

The LiDARs used in the KITTI dataset are mechanical spinning LiDARs with 360-degree coverage. These LiDARs have a high point cloud density, but when the point cloud is matched for that image, they are sparse, as shown in Figure 5, and depth image using these sparse data is challenging to use. Therefore, by increasing the size of the projected point cloud, this sparsity should be lowered. Figure 6 is the depth image depending on the different sizes of point cloud.

### 3.2. Scattering Coefficient Estimation

To obtain a transmission image for dehazing from depth image, a scattering coefficient is required. However, it is not easy to obtain an accurate scattering coefficient with only the image obtained from camera. Therefore, using synthetic haze image and ground truth image, a model that can estimate the scattering coefficient should be obtained.

The synthetic haze images required for this were synthesized based on the atmospheric scattering model using the KITTI dataset [39] and depth images. The depth image used for the synthesis was generated by monodepth2 [40].

**Figure 7:**
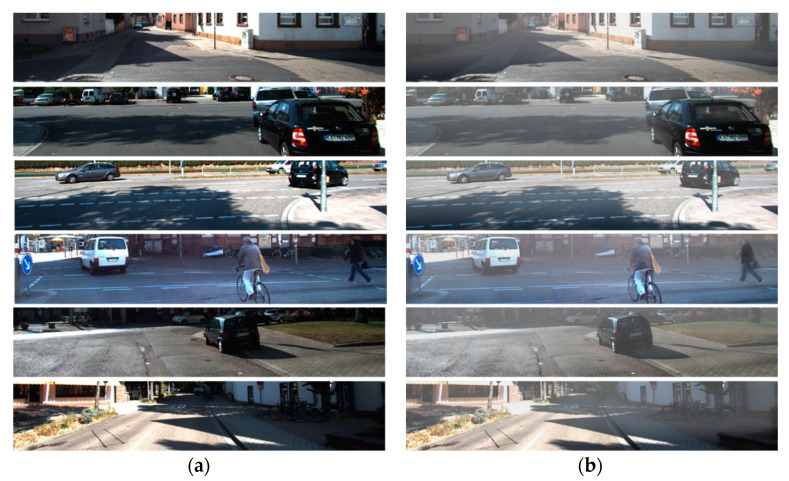
Synthetic haze image with ground truth: (**a**) ground truth; (**b**) synthetic haze image.

**Figure 8:**
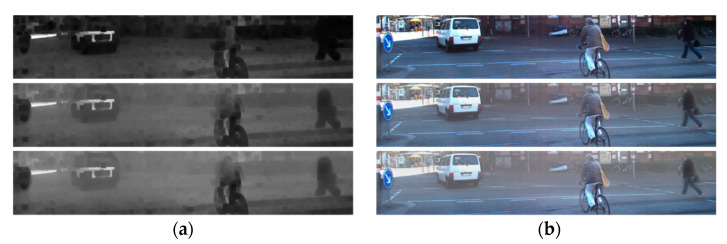
Generated dark channel depending on different intensity of haze: (**a**) dark channel; (**b**) hazy image.

Figure 7 shows that haze is generated throughout the images. Thus, estimating the dark channel of hazy image and calculating average brightness is higher than when calculated in a no-haze situation. This can be confirmed in Figure 8. Such a relationship allows us to model equations that obtain the scattering coefficient from the dark channel’s average brightness. In this study, this average brightness is called the dark channel means (DCMs). To model the equation, an optimal scattering coefficient value for the haze image should be obtained. This can be obtained by performing dehazing of each value of the scattering coefficient, gradually increasing the scattering coefficient, and comparing the obtained results with the ground truth. Comparison of images is performed by calculating the mean square error (MSE) for pixels of each image, and when the mean square error becomes the smallest, the value at that time is set as the optimal scattering coefficient.

Algorithm 1 is pseudocode for estimating optimal scattering coefficients. The input data of the algorithm is ground truth image (GT), hazy image (hazy), and depth image (depth) (line 3). The algorithm initializes the scattering coefficient to 0 and 0.01 (line 1 and 2), incrementally increases them (line 16 and 17), and dehazing is performed using Equation (2) (line 10 and 11) and Equation (3) (line 7 and 8).

After dehazing, MSE is obtained through the dehazed image and GT (line 13 and 14). When MSE becomes the smallest (line 5), the scattering coefficient is determined as the optimal scattering coefficient (line 20).

**Figure 9:**
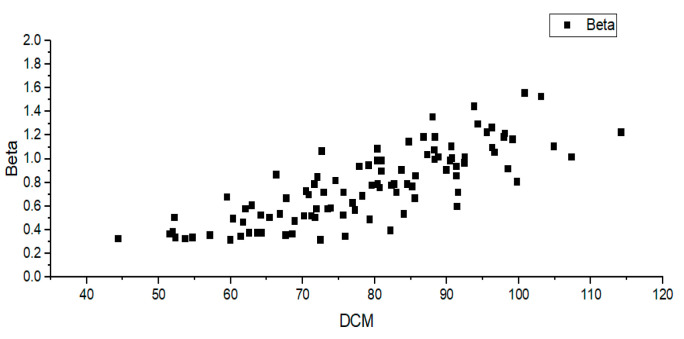
Distribution of DCM-optimal scattering coefficient.

Using the method in Algorithm 1, the optimal scattering coefficient for each hazy image is estimated. Next, we obtain the DCM of each hazy image and create a distribution chart using the DCM and the optimal scattering coefficient. The following Figure 9 refers to a scattering coefficient—DCM distribution chart obtained by the synthetic KITTI haze dataset. In Figure 9, the x-axis represents the DCM, and the y-axis represents the scattering coefficient.

**Figure 10:**
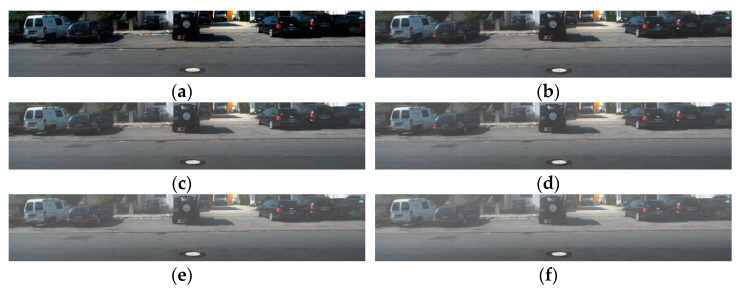
Synthetic image generated from difference of scattering coefficient: (**a**) β = 0; (**b**) β = 0.001; (**c**) β = 0.0015; (**d**) β = 0.002; (**e**) β = 0.0025; (**f**) β = 0.003.

A total of 100 synthetic haze images were used to obtain the scattering coefficient model. This is the result of synthesizing 20 types of images in 5 stages depending on th level of haze generation. Figure 10 shows synthetic haze images with varying scattering coefficients of step 5.

Using the distribution of the DCM-optimal scattering coefficient for 100 hazy synthetic images, the relationship between the two variables can be derived.

(11) β=0.0174∗DCM−0.5919

Equation (11) is a model obtained by linear regression of the DCM-optimal scattering coefficient distribution for 100 synthetic images.

**Algorithm 1** Estimate β1: 
$\beta_{1}=0$
2: 
$\beta_{2}=0.01$
3: 
**Input**: GT, hazy, depth
4:  **For** ${\mathrm{MSE}}_{1}<{\mathrm{MSE}}_{2}$ **do**
5:          ${\mathrm{trans}}_{1}=\mathrm{transmission}(\beta_{1},\mathrm{depth})\ldots \ldots eq.(3)$
6:          ${\mathrm{trans}}_{2}=\mathrm{transmission}(\beta_{2},\mathrm{depth})\ldots \ldots eq.(3)$
7:         
${\mathrm{dehaze}}_{1}={\mathrm{dehazing}(\mathrm{hazy},\mathrm{trans}}_{1})\ldots \ldots eq.(2)$
8:          ${\mathrm{dehaze}}_{2}={\mathrm{dehazing}(\mathrm{hazy},\mathrm{trans}}_{2})\ldots \ldots eq.(2)$
9:          ${\mathrm{MSE}}_{1}={\mathrm{MSE}(\mathrm{dehaze}}_{1},\mathrm{GT})\;$
10:        ${\mathrm{MSE}}_{2}={\mathrm{MSE}(\mathrm{dehaze}}_{2},\mathrm{GT})\;$
11:         $\beta_{1}=\beta_{1}+0.01$
12:         $\beta_{2}=\beta_{2}+0.01$
13: **end for**
14: return $\beta_{1}-0.01$

### 3.3. Transmission Image Refine

The transmission image of the atmospheric scattering model can be obtained by Equation (3). The raw transmission image is estimated using the depth image and the scattering coefficient, obtained by point cloud 2D projection and Equation (11), respectively.

**Figure 11:**
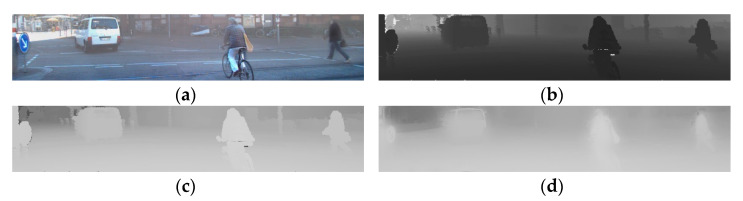
Refine transmission image: (**a**) hazy image; (**b**) grayscale depth image generated from point cloud projection; (**c**) raw transmission image from grayscale depth image; (**d**) refined transmission image.

The raw transmission image is estimated as shown in Figure 11c. Since the transmission image is generated from the depth image via point cloud 2D projection, the raw transmission image shows the block effect in He et al. [26]. Therefore, the raw transmission image obtained through Equation (3) should be refined. To refine the transmission image, the hazy image and raw transmission image are used, and the guided filtering is performed.

### 3.4. Background Parameter

**Figure 12:**
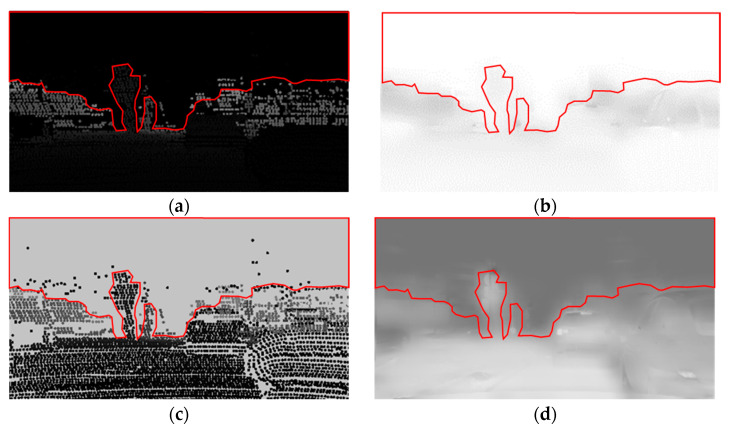
Comparison applying background parameter: (**a**) before applying; (**b**) transmission image generated from (**a**); (**c**) applying background parameter; (**d**) transmission image generated from (**c**).

Background parameters are used to prevent dehazing performance degradation due to differences in detection range between camera and LiDAR. If an object can be seen from the camera, but is outside the detection range of LiDAR, the pixel value of the transmission image is 1 for the absence of point cloud, so dehazing is not effective. Therefore, for places where point cloud does not exist, it should be set to a value between the maximum pixel value that point cloud can have and the original maximum pixel value of 255. The compensation process for an empty space in which the point cloud does not exist is performed through a background parameter. Figure 12 is the result of the transmission image after applying the background parameter.

**Figure 13:**
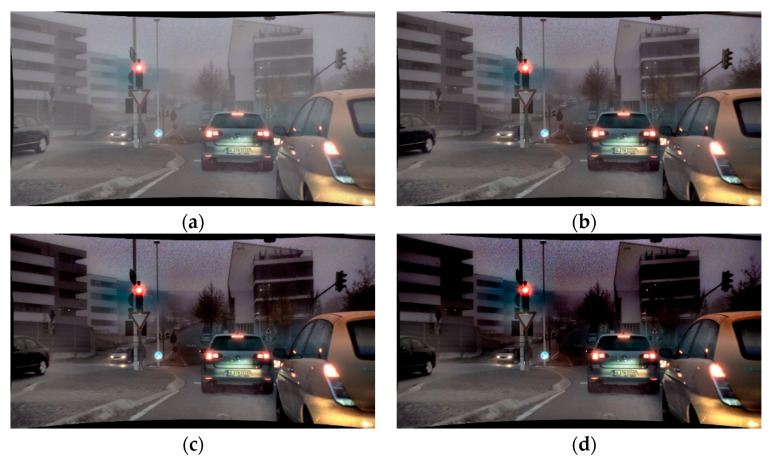
Dehazed results at various background parameters: (**a**) hazy input; (**b**) 120; (**c**) 195; (**d**) 255.

The background parameter was set to 195 because it was the most effective after several times of dehazing through real-world haze photographs. It is impossible to set the parameter through quantitative analysis because there is no ground truth image for the actual haze occurrence image. Therefore, the background parameter was set by a heuristic approach. Figure 13 is the result of dehazing through several background parameters.

### 3.5. Scene Radiance Recovery

#### 3.5.1. Estimation of Atmospheric Light

Generally, the appropriate value for atmospheric light in the hazy image would be the strongest pixel value within the image. In this case, however, it has the disadvantage of not being able to distinguish white objects. To compensate for these shortcomings, the dark channel prior is used. After obtaining the dark channel prior from the hazy image, the top 0.1% of the brightest pixels are drawn from the dark channel. We can consider these pixels as the most hazy pixels. So, among these pixels, the brightest pixel in the input image I(x), is selected as the atmospheric light A(x).

#### 3.5.2. Dehazing Process

The 2D depth image is obtained through projection of the point cloud, and the scattering coefficient is obtained through the DCM—scattering coefficient equation. Transmission images can then be obtained through the acquired depth image and the scattering coefficient. Thus, scene radiance recovery can be performed through the atmospheric scattering model.

(12) $$J(x)=\frac{I(x)-A(x)}{t(x)}+A(x)$$

To avoid noise generation due to the transmission image, it is necessary to set the lower bound of the transmission image. The equation in which the lower bound is added can be expressed as follows.

(13) $$J(x)=\frac{I(x)-A(x)}{\max\{t(x),0.1\}}+A(x)$$

## 4. Simulation

The dehazing algorithm was written in Python, and simulations performed on the Intel i5-3470@3.20GHz, 8GB RAM. In simulation, the improvement of the image was determined by comparing the mean square error (MSE), peak signal-to-noise ratio (PSNR), image enhancement factor (IEF), and structural similarity index measure (SSIM) [41] of the hazy image and dehazed image. In addition, our proposed algorithm was quantitatively and qualitatively compared to existing algorithms, such as He et al. [26], Tan et al. [28], and Fattal et al. [29].

### 4.1. Quantitative Analysis of Dehazing Improvement Quality

**Figure 14:**
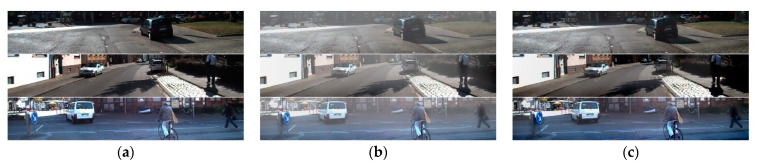
Hazy image with ground truth and dehazed image: (**a**) ground truth; (**b**) hazy image (c) dehazed image.

Dehazing improvement performance of the proposed algorithm is quantitatively analyzed using performance improvement parameters. When the synthetic haze image is composed, β is set to 0.003 and atmospheric light is set to 210. Figure 14b is the synthetic haze image and Figure 14c is the result of dehazing.

Performance analysis of three pairs of hazy images and dehazed images was performed via PSNR, SSIM, and MSE. First, performance analysis for hazy image and ground truth image is shown in GT-Hazed in Table 1, Table 2 and Table 3 and performance analysis for ground truth and the dehazed image is shown in GT-Dehazed in Table 1, Table 2 and Table 3. The PSNR and SSIM of GT-Dehazed were higher than those of GT-Hazed, and the MSE of GT-Dehazed were lower than that of GT-Hazed. Therefore, the analysis results from the image analysis parameters show that all three images have been improved.

### 4.2. Quantitative Comparison of Different Dehazing Algorithm

**Figure 15:**
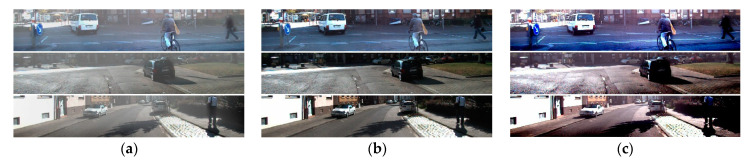

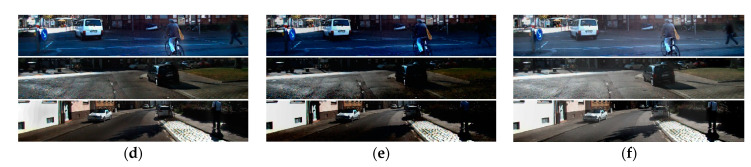
Dehazing result of various algorithms: (**a**) hazy image; (**b**) ground truth; (**c**) Fattal’s method; (**d**) He’s method; (**e**) Tan’s method; (**f**) proposed method.

We performed a quantitative performance analysis between the existing dehazing algorithm and the proposed algorithm. Existing algorithms used for comparison of performance are Tan et al. [28], Fattal et al. [29], and He et al. [26]. Analysis of the resulting images was conducted via MSE, PSNR, IEF, and SSIM as shown in Table 4, Table 5 and Table 6. Figure 15 shows an input image, synthetic haze image, and a dehazing image generated from the proposed and existing algorithms, respectively.

The proposed algorithm is designed to make the MSE smallest. Thus, if the DCM of the hazy image input does not deviate significantly from the model of DCM and scattering coefficient, the proposed method achieves the smallest MSE of the four methods. In addition, as MSE became smaller, other performance parameters were improved.

## 5. Experiments

Based on the proposed algorithm, we performed dehazing using the DENSE dataset [42] from Ulm University in case of natural fog.

### 5.1. Comparison of Dehazing Results

**Figure 16:**
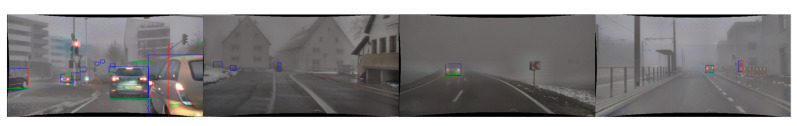
Ground truth of object detection.

**Figure 17:**
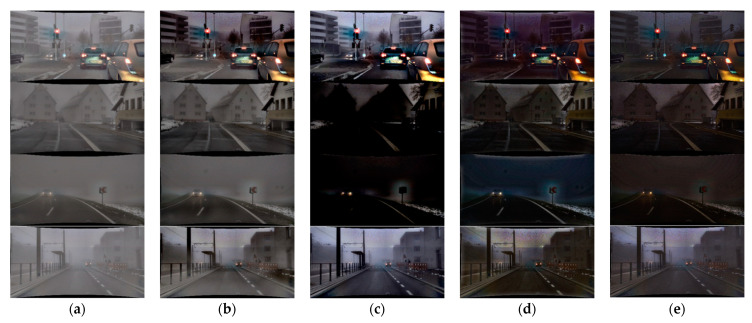
Results of various algorithms: (**a**) hazy input; (**b**) proposed; (**c**) Fattal; (**d**) He; (**e**) Tan.

**Figure 18:**
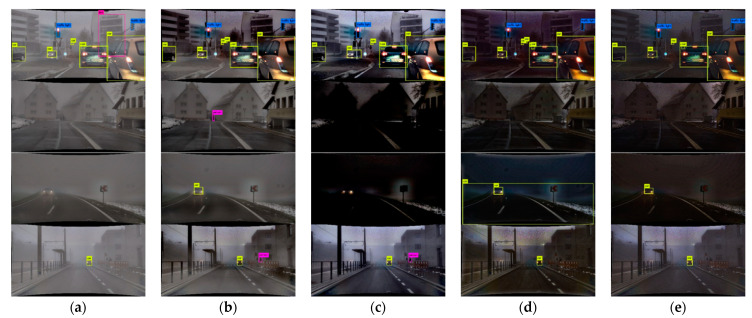
Qualitative comparison of various algorithms: (**a**) hazy input; (**b**) proposed; (**c**) Fattal; (**d**) He; (**e**) Tan.

Ground truth of object detection is as shown in Figure 16. Figure 17 shows the results of the proposed and existing algorithms and Figure 18 shows the results of qualitative evaluation with YOLO V3 using dehazed results. We confirmed that dehazing was correctly performed under real haze and fog conditions through the dataset. In addition, through YOLO V3 object detection, we also confirmed that the image was improved after dehazing.

### 5.2. Time Consumption

We compared computing time for 1920 × 1024 pixel images. The algorithms were written in Python and performed on the Intel i5-3470 CPU @ 3.2Ghz, 8GB RAM. The proposed algorithm took approximately 0.47 s per image. He’s algorithm took about 0.4 s, Tan’s algorithm took about 0.2 s, and Fattal’s algorithm took about 75 s. Table 7 represents the progressing time and frame per second (FPS).

## 6. Conclusions

We present a method for performing dehazing via LiDAR depth image and DCM-scattering coefficient model. The proposed algorithm obtains the scattering coefficient model through the DCM and scattering coefficient relationship. Dehazing is then performed through the scattering coefficient and point cloud projection depth image obtained from LiDAR. Through simulations, we confirmed that the dehazed image is obtained effectively. In the simulation, MSE showed improvement over conventional algorithms, and PSNR and IEF, which are dependent on MSE, have also shown improvements. Furthermore, SSIM, an important parameter used in image recognition, showed an average improvement of about 24% over conventional algorithms.

However, the proposed algorithm has a problem to solve. First, when estimated using DCM, the scattering coefficient was able to perform dehazing effectively on most haze images, but using only pixel value mean may be unreliable. If there are many colorful objects in the near distance, the DCM can still be low, even with much haze. Consequently, it will deviate from the scattering coefficient estimation model, which results in dehazing being ineffective. Such problems of DCM could be addressed by CNN and by supervised learning for image and effective scatter coefficient.

In addition, because the depth image is obtained through LiDAR, dehazing may not work effectively if LiDAR malfunctions. We will improve these existing problems through further research. Moreover, there is a real-time problem. As of now, there are difficulties in operating in real time. This occurs because the imaging operation is performed simply with CPU only. Therefore, it is planned to secure real time by making it possible to operate in parallel through GPU operations through future research.

## Figures and Tables

**Table 1:** Quantitative analysis of improvement using first row image from Figure 14.

	GT-Hazed	GT-Dehazed	Improvement (%)
PSNR (dB)	14.68	24.44	66.43
SSIM	0.8349	0.9658	15.68
MSE	0.0341	0.0036	−89.41

**Table 2:** Quantitative analysis of improvement using second row image from Figure 14.

	GT-Hazed	GT-Dehazed	Improvement (%)
PSNR (dB)	13.14	25.19	91.63
SSIM	0.7373	0.9525	25.48
MSE	0.0485	0.0031	−93.81

**Table 3:** Quantitative analysis of improvement using third row image from Figure 14.

	GT-Hazed	GT-Dehazed	Improvement (%)
PSNR (dB)	15.41	23.866	54.92
SSIM	0.8066	0.8502	5.410
MSE	0.0288	0.0041	−85.76

**Table 4:** Comparison result 1 using upper image from Figure 15.

	PSNR (dB)	IEF	SSIM	MSE
Fattal et al.	16.56	1.542	0.8033	0.0221
He et al.	17.60	1.955	0.8711	0.0174
Tan et al.	14.00	0.8543	0.6079	0.0398
Proposed	24.44	9.451	0.9658	0.0036

**Table 5:** Comparison result 2 using middle image from Figure 15.

	PSNR (dB)	IEF	SSIM	MSE
Fattal et al.	15.86	1.867	0.7113	0.0260
He et al.	17.14	2.508	0.9073	0.0193
Tan et al.	14.00	1.219	0.6601	0.0398
Proposed	25.19	16.01	0.9252	0.0030

**Table 6:** Comparison result 3 using lower image from Figure 15.

	PSNR (dB)	IEF	SSIM	MSE
Fattal et al.	17.05	1.461	0.6803	0.0197
He et al.	18.19	1.900	0.8828	0.0152
Tan et al.	15.10	0.9326	0.6314	0.0309
Proposed	23.87	7.015	0.8502	0.0041

**Table 7:** Time consumption of each algorithms.

	Proposed	He *	Tan	Fattal
Time (s)	0.47	0.4	0.2	75
FPS	2.1	2.5	5	0.013

* Guided filter used (not soft matting).
